# Integrated Isoform Sequencing and Dynamic Transcriptome Analysis Reveals Diverse Transcripts Responsible for Low Temperature Stress at Anther Meiosis Stage in Rice

**DOI:** 10.3389/fpls.2021.795834

**Published:** 2021-12-17

**Authors:** Zhaojun Qu, Yan Jia, Yuyang Duan, Hongyang Chen, Xinpeng Wang, Hongliang Zheng, Hualong Liu, Jingguo Wang, Detang Zou, Hongwei Zhao

**Affiliations:** Key Laboratory of Germplasm Enhancement, Physiology and Ecology of Food Crops in Cold Region, Ministry of Education, Northeast Agricultural University, Harbin, China

**Keywords:** rice, anther, low-temperature stress, carbon and nitrogen metabolism, WGCNA

## Abstract

Low temperatures stress is one of the important factors limiting rice yield, especially during rice anther development, and can cause pollen sterility and decrease grain yield. In our study, low-temperature stress decreased pollen viability and spikelet fertility by affecting the sugar, nitrogen and amino acid contents of anthers. We performed RNA-seq and ISO-seq experiments to study the genome-wide transcript expression profiles in low-temperature anthers. A total of 4,859 differentially expressed transcripts were detected between the low-temperature and control groups. Gene ontology enrichment analysis revealed significant terms related to cold tolerance. Hexokinase and glutamate decarboxylase participating in starch and sucrose metabolism may play important roles in the response to cold stress. Using weighted gene co-expression network analysis, nine hub transcripts were found that could improve cold tolerance throughout the meiosis period of rice: Os02t0219000-01 (interferon-related developmental regulator protein), Os01t0218350-00 (tetratricopeptide repeat-containing thioredoxin), Os08t0197700-00 (luminal-binding protein 5), Os11t0200000-01 (histone deacetylase 19), Os03t0758700-01 (WD40 repeat domain-containing protein), Os06t0220500-01 (7-deoxyloganetin glucosyltransferase), Pacbio.T01382 (sucrose synthase 1), Os01t0172400-01 (phospholipase D alpha 1), and Os01t0261200-01 (NAC domain-containing protein 74). In the PPI network, the protein minichromosome maintenance 4 (MCM4) may play an important role in DNA replication induced by cold stress.

## Introduction

Rice (*Oryza sativa* L.) is a staple food for over 60% of the Chinese population (Zhu et al., 2010). Rice growth responds differently to low temperature (LT) stress during different developmental stages. Studies have shown that the most sensitive period in the reproductive stage is the transition of the tetrad to the early uninucleate stage, that is, the meiosis stage (Sharma and Nayyar, 2016). The most evident morphological changes with LT stress are the reduction of anther dehiscence, pollen germination, pollen viability, pollen tube growth, and number of pollen grains (Coast et al., 2016; Susanti et al., 2019). LT stress during this period disrupts the callose wall formed in tapetal cells and limits the provision of nutrition to young microspores and developing pollen grains, leading to male sterility (Oliver et al., 2005; Mamun et al., 2006, 2010; Oda et al., 2010; Fahad et al., 2018; Zhang et al., 2021).

Pollen development requires sufficient carbohydrates (Hirsche et al., 2017). Polysaccharides are transported from the tapetum to pollen for starch synthesis, and provide energy for pollen tube germination (Mamun et al., 2006). Studies have shown that the reduction of starch content in pollen grains decreases pollen viability (Lee et al., 2020). Previous research found that LT stress increased abscisic acid (ABA) levels, decreased gibberellic acid (GA) levels in the tapetum, and decreased the activity of cell wall-bound acid invertase, thereby inducing tapetum hypertrophy and delaying programmed cell death (PCD), leading to sucrose accumulation in the tapetum, and disrupted hexose production and starch formation in the pollen grains, and resulting in a decline in pollen viability (Oliver et al., 2005, 2007; Li et al., 2006; Zhang et al., 2021).

Recent studies have shown that male sterility is linked to amino acid metabolism in anthers (Qu et al., 2014). The reduced synthesis of amino acids is accompanied by abnormal tapetum development (Fang et al., 2016). Amino acid and proline metabolism at the transcription level in male reproductive tissues is essential for pollen development and viability (Ji et al., 2018; Kiran et al., 2021). The anthers and pollen both also accumulate and store nitrogen, which plays a role in their protection under temperature stress (Santiago and Sharkey, 2019).

In terms of genes, previous studies have found that knockouts or mutations of OsINV4 (cell wall invertase), RA68 (AY568677 in the DNA databank), OsHXK5 (hexokinase), OsPGM (phosphoglucomutase), and OsUGP2 (UDP-glucose pyrophosphorylase) reduce the starch content of the anther, which ultimately leads to a decrease in spikelet fertility (Oliver et al., 2005; Kong et al., 2007; Mu et al., 2009; Li T. et al., 2010; Lee et al., 2020). The development of male gametophytes is severely impaired in *Arabidopsis* P5CS1 and P5CS2 proline-deficient mutants (Mattioli et al., 2012). The AP2/ERF, bHLH, bZIP, NAC, WRKY, and MYB transcription factor families play an important regulatory roles in the response of rice to LT stress (Li F. et al., 2010; Bai et al., 2015; Kim C. Y. et al., 2016; Chang et al., 2017; Guo et al., 2018; Ritonga et al., 2021). However, these earlier reports had focused on the leaves of seedlings, and few studies have investigated the molecular mechanism of the rice anther response to LT stress during meiosis.

Nowadays, LT stress of rice is a worldwide concern that has been seriously harmed 24 countries, especially in northeast China, Korea, and Japan during the reproductive stage (Xia et al., 2016; Zhang et al., 2017). Heilongjiang, located in the northernmost part of China, is a cold temperate climate region, with large annual temperature fluctuations often resulting in LT stress during the reproductive stage of rice (July to August). In this study, we performed RNA-seq and ISO-seq experiments to study the genome-wide transcript expression profiles of cold-stressed anthers from tolerant cultivars. Candidate transcripts and pathways involved in cold-stress responses were identified, and we demonstrated that DN428 improves the cold tolerance of rice at the reproductive stage by mediating ABA, ubiquitination, and glutathione and sugar metabolism, indicating that these transcripts can improve cold tolerance throughout the reproductive growth period of rice.

## Materials and Methods

### Plant Materials and Cold Treatments

The experiment was conducted at Northeast Agricultural University, Heilongjiang Province, China during the rice growing season of 2020. Rice variety DN428 was chosen for analyses. It was screened out from a set of 110 varieties, and demonstrated high spikelet fertility and pollen vitality at 17°C LT stress during the reproductive stage. This is a critical cold stress temperature for rice in the reproductive stage (Yan et al., 2020).

DN428 was raised in the seedbed with a sowing date of April 10th and a transplantation date of May 15th in plastic pots (diameter 30 cm, height 28 cm) in a greenhouse (FYS-20, Nanjing, China), at 28°C and 22°C (day and night, respectively), under a 12-h light/dark photoperiod, and with a relative humidity of 80%. Growth took place on four hills. On each hill, 150 pots were planted, with 3 plants in each pot. When the auricle of the flag leaf was approximately 3–5 cm below the auricle of the penultimate leaf on the plants, this was an indicator that pollen development should be in the meiosis stage (Oliver et al., 2005). This was when the LT treatment began. The pots were moved into a 17°C low-temperature greenhouse at 8 am and placed into five 30-pot groups, which were given LT treatment for either 0 (D0),1 (D1), 2 (D2), 3 (D3), or 4 (D4) days. All treatment groups were prepared in triplicate. After the end of LT stress, each treatment group was returned to the normal greenhouse. The two greenhouses were managed under the same environmental conditions with the exception of temperature.

### Determination of Physiological Related Indicators

In this study, anthers were taken from the upper third of the panicle, in triplicate, placed at 105°C for 30 min, and then dried to a constant weight at 80°C. These dry anthers were taken to determine the physiological index at the end of each treatment. The concentrations of soluble sugars and starch were determined using anthranone-H_2_SO_4_ colorimetry (Dien et al., 2019). Sucrose and fructose content were measured using the resorcinol method (Du et al., 2020). Glucose content was measured using a glucose oxidase-peroxidase assay kit (Suzhou Grace Biotechnology Co., Ltd. Jiangsu, China). The nitrate content was measured using salicylic acid colorimetry (Cataldo et al., 1975). The ammonium concentration was measured using the ninhydrin method (Huang et al., 2018). Amino acids concentrations were measured using high-performance liquid chromatography (Dahl-Lassen et al., 2018). Total carbon (C) and nitrogen (N)content were measured using a Primacs SNC-100 C/N analyzer (Skalar Analytical B.V., Breda, Netherlands) (Pinto et al., 2020).

Pollen was taken from the upper third of the anther of fresh panicle at the flowering stage, in triplicate, stained with 1% potassium iodide solution (I_2_-KI), and observed under a microscope (Eclipse-80i, Nikon, Japan). Pollen viability was calculated as the percentage of blue pollen grains visible in the total pollen grains (Zhou et al., 2011). At the mature stage, four hills of panicles were taken for each treatment, in triplicate, and the grain number and empty grains of primary and secondary branches were counted. These data were then used to calculate the total spikelet fertility and spikelet fertility of the primary and secondary branches.

### Statistical Analysis

Data analyses were conducted using SPSS 22.0 (SPSS, Chicago, United States). Duncan’s multiple range test was performed to compare the mean differences at *P* < 0.05. Graphs were drawn using Origin 2020 (Origin Lab, California, United States).

### PacBio Iso-Seq and RNA-Seq Library Preparation and Sequencing

Total RNA from each fresh anther sample was extracted using a TRlzol Reagent kit (Life Technologies, California, United States), according to the manufacturer’s instructions.

A Clontech SMARTer PCR cDNA Synthesis Kit was used to synthesize cDNA from RNA samples used for PacBio sequencing, according to the manufacturer’s instructions. The amplified cDNA products were used to generate the SMRTbell template libraries according to the Iso-Seq protocol. Libraries were prepared for sequencing by annealing a sequencing primer and adding polymerase to the primer-annealed template. The polymerase-bound templates were bound to MagBeads and six SMRT cells were used for sequencing the poly (A) + library on a PacBio RS II sequencer.

A total of 1 μg of RNA per sample was used as the input material to generate sequencing libraries using the NEBNext Ultra RNA Library Prep Kit for Illumina (NEB) according to the manufacturer’s instructions. The libraries were sequenced on an Illumina HiSeq X Ten platform, and 150 bp paired-end reads were generated.

### PacBio and Illumina Data Analysis

The SMRT Analysis software package (v3.0, Pacific Biosciences) was used for the Iso-Seq data analysis (Gonzalez-Garay, 2016). Transcripts were annotated by conducting blastx searches against the SwissProt database, with an E-value threshold of 0.00001 (Bairoch and Apweiler, 2000).

Adaptor sequences, unknown nucleotides > 5%, and reads with sequencing error rates < 1% were removed using a Perl script. Then, the clean reads were mapped to the *Oryza sativa* reference genome with the Iso-Seq update using TopHat2 software (v. 2.1.1) (Kawahara et al., 2013; Kim et al., 2013). Transcript expression levels were estimated using FPKM values (fragments per kilobase of transcript per million fragments mapped) using the HTseq software (v. 0.11.1) (Anders et al., 2015). Differential expression analysis between the two conditions/groups was performed using the DESeq 2 R package (1.10.1) (Love et al., 2014). The resulting P values were adjusted using the Benjamini-Hochberg approach to control the false discovery rate. Genes identified by DESeq2 with FDR ≤ 0.01 and | FC| ≥ 2 were defined as differentially expressed. GO enrichment analysis was performed using topGO with Fisher’s exact test, and negative log10 transformed P values were visualized using pheatmap (Alexa and Rahnenführer, 2009). The WGCNA (v1.42) package in R was used to construct the co-expression networks (Langfelder and Horvath, 2008). The protein–protein interactions (PPI) of the differentially expressed genes (DEGs) were predicted using the STRING database of rice PPI data (Smoot et al., 2011; Szklarczyk et al., 2021).

### Validation of Differentially Expressed Genes by RT-PCR

A total of 16 DEGs (Supplementary Table 1) were selected for validation by quantitative reverse transcription polymerase chain reaction (qRT-PCR). RNA was extracted from the samples and reverse transcribed into cDNA using the HiFiScript cDNA Synthesis Kit (CWBIO, Beijing, China), according to the manufacturer’s instructions. The qRT-PCR was performed with SYBR Green I dye (Osaka, Japan) on a LightCycler 96 system (Roche, Basel, Switzerland). PCR amplification was conducted with an initial step at 95°C for 5 min followed by 45 cycles of 15 s at 95°C, 25 s at 56°C, and 35 s at 72°C. The qRT-PCR results were evaluated using the 2^–ΔΔCT^ method (Shannon et al., 2003). Each candidate gene was detected in triplicate and expressed as the mean value ± standard error. The reference gene used was Actin1. Each sample contained three biological replicates and three technical replicates.

## Results

### Physiological Responses of Anther Under Low-Temperature Stress in Rice

**FIGURE 1:**
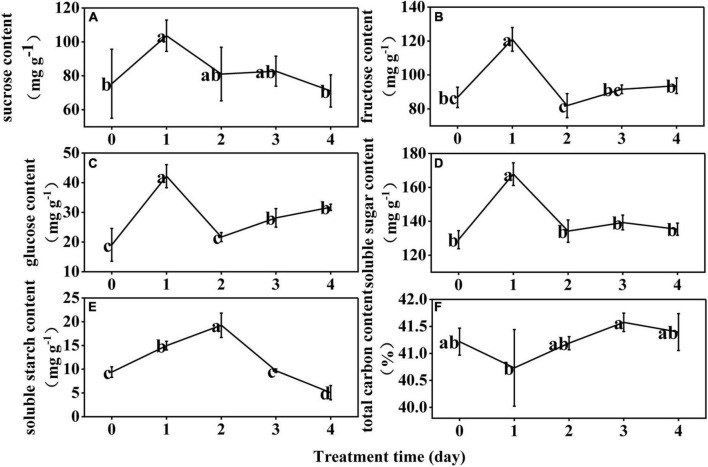
Effect of low temperature on the sucrose **(A)**, fructose **(B)**, glucose **(C)**, soluble sugar **(D)**, soluble starch **(E)**, and total carbon **(F)** content in the anther of rice. The small letters represent significant differences of comparison between the five groups.

At D1, the sucrose, fructose, glucose, and soluble sugar contents of the rice anthers increased by 38, 39, 121, and 30%, respectively. At D2, they decreased by 22, 32, 49, and 20%; and then persistently decreased throughout D3 and D4 (Figures 1A–D). The soluble starch content continued to increase significantly at D1 and D2, and decreased significantly at D3 and D4 (Figure 1E). The total carbon content was not significantly different from controls through the LT treatment (Figure 1F).

**FIGURE 2:**
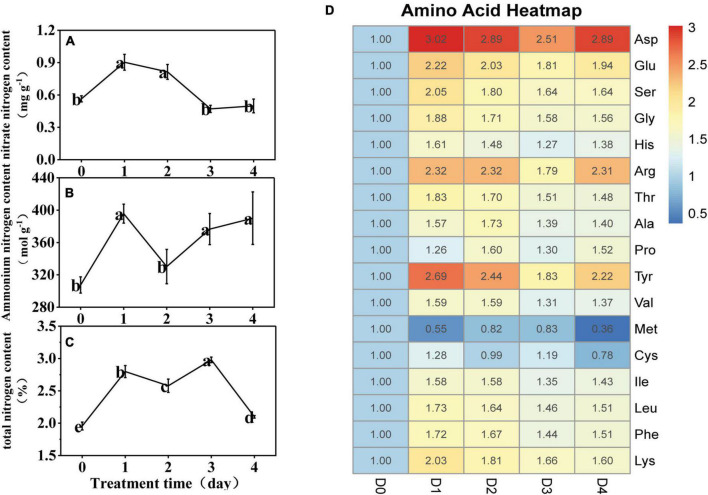
**(A–C)** Effect of low temperature on the nitrate nitrogen, ammonium nitrogen, and total nitrogen content in the anther of rice. The small letters represent significant differences of comparison between the five groups. **(D)** Heatmap of the 17 amino acid content under low temperature stress. The value represents the ratio of the amino acid content to the D0.

Compared with D0, the nitrate nitrogen increased significantly at D1 and D2, and insignificantly at D3 and D4 (Figure 2A). The ammonium nitrogen content increased by 29% at D1, and then significantly decreased by 17% at D2; however, by D3 and D4, it increased again to the D1 level (Figure 2B). The total nitrogen content increased significantly during LT treatment (Figure 2C). Under LT stress, concentrations of 15 amino acids increased, especially Asp, Glu, Arg, and Tyr (which increased by an average of 2.8, 2.0, 2.2, and 2.3 times, respectively). Met persistently decreased significantly, and Cys decreased significantly at D4 (Figure 2D).

**FIGURE 3:**
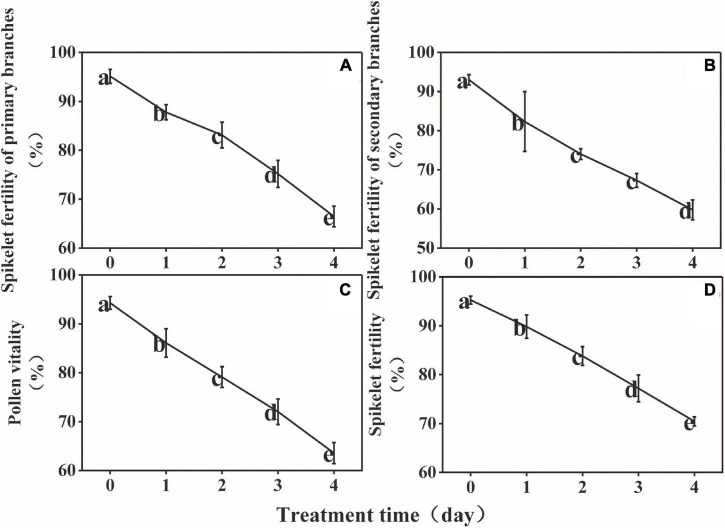
Effect of low temperature on the spikelet fertility of the primary branches **(A)**, spikelet fertility of the secondary branches **(B)**, pollen vitality **(C)**, and spikelet fertility **(D)** of rice. The small letters represent significant differences of comparison between the five groups.

The spikelet fertility of the primary branches, spikelet fertility of the secondary branches, overall spikelet fertility, and pollen vitality decreased persistently during LT treatment from 95, 93, 94, and 95%, respectively, at D0, to 66, 60, 63, and 70%, respectively, at D4 (Figure 3). The results of multiple linear stepwise regression indicated that Pro, Ala, Asp, soluble starch content, and total nitrogen content of the anthers could be used as a comprehensive evaluation index of spikelet fertility and pollen vitality for rice (Supplementary Table 2). The decision coefficient of Pro was the highest (*R*^2^ = 0.488), which was the main positive determinant of spikelet fertility (Supplementary Table 3). The decision coefficient of Ala was the smallest (*R*^2^ = −8.96), which was the limiting factor for pollen vitality (Supplementary Table 4).

### Sequencing of the Rice Transcriptome Using the PacBio Iso-Seq Platform

**FIGURE 4:**
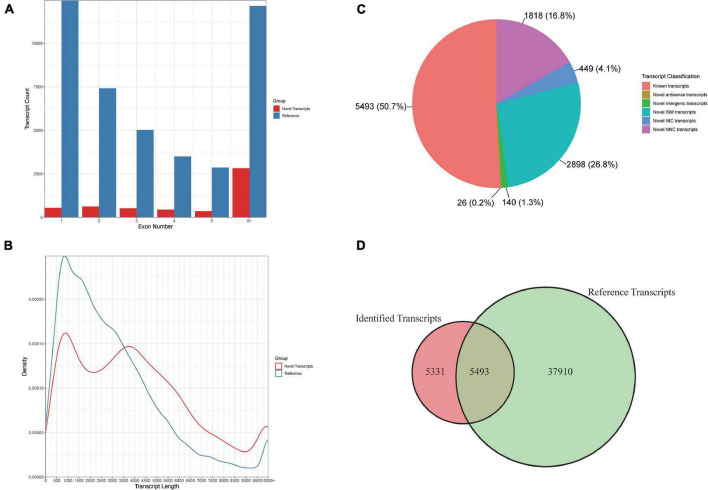
Comparison of rice genome IRGSP-1.0 (Reference) and PacBio Iso-Seq data isoform annotations. **(A)** Distribution of the percentage transcripts with different exon numbers for reference and PacBio Iso-Seq data. **(B)** Comparison of transcripts model and PacBio Iso-seq isoform length. **(C)** The percentage of PacBio Iso-Seq isoforms that are the same as existing gene models, and novel isoforms. **(D)** Comparison of gene model and PacBio Iso-seq isoform number.

To identify the diverse transcripts that regulate the cold response in rice anthers, we sequenced the transcriptomes of anthers using the PacBio Iso-seq (isoform sequencing) platform. This platform provided long reads of full-length transcripts. To identify the longest transcripts possible, high-quality RNA was used for sequencing. Total RNA was extracted from the rice anther sample, which was mixed with D0–D4. The library was sequenced on the PacBio Sequel instrument using sequencing kit 2.1 (Pacific Biosciences) with three SMRT cells, yielding 3,79,496 Reads of Inserts (ROI), of which 2,29,295 (60.4%) were full-length [containing the 5′ barcoded primer, 3′barcoded primer, and poly (A) tail] (Figures 4A,B). ToFu (Gordon et al., 2015) processing yielded 10,824 non-redundant isoforms in rice. Isoforms identified from Iso-Seq were longer than those in the reference database, and more exons were found in this study.

We compared these 10,824 isoforms against the *Oryza sativa* genome (Figures 4C,D). We found 5,331 new isoforms, and classified them into five groups as follows: (i) 2,898 incomplete splice match (ISM) transcripts, (ii) 449 novel in catalog (NIC) transcripts, (iii) 1,818 novel not in catalog (NNC) transcripts, (iv) 26 novel antisense transcripts, and (v) 140 novel intergenic transcripts. The high percentage of new isoforms demonstrates that PacBio full-length sequencing can provide a comprehensive set of isoforms.

### Transcripts Differentially Expressed in Cold-Stressed Anthers at Different Timepoints

After filtering out adapter and low-quality sequences from the RNA-seq libraries obtained from LT-treated anther tissues, approximately 95.2 Gb of clean bases were obtained in the 15 transcriptome libraries, with a quality score of Q30 ≥ 92%. Salmon was used to quantify transcript abundance from the RNA-seq reads. Transcripts per million (TPM) was used to quantify transcript expression levels. The Pearson correlation coefficient between biological replicates, was more than 0.83, revealing a good correlation between the three biological replicates of the different samples (Supplementary Figure 1).

**FIGURE 5:**
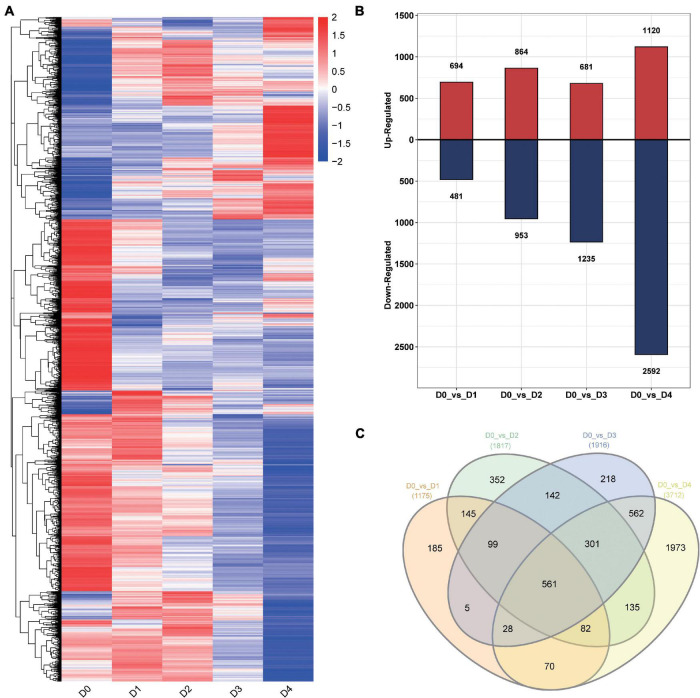
Transcripts differentially expressed in cold-stressed anthers at different timepoints. **(A)** Heat map of expression level for 4,859 DETs. **(B)** Histogram of the DETs between low-temperature groups and control group from different timepoints and the control group. **(C)** DETs between low-temperature groups from different timepoints and the control group.

For differential expression transcript analysis, stringent criteria (fold-change ≥ 2 and Q-Value ≤ 0.01) were used to select differentially expressed transcripts (DETs). A total of 4,859 DETs between the LT groups from different times and the control group (D0 vs. D1, D0 vs. D2, D0 vs. D3, and D0 vs. D4) were identified (Figure 5A). A total of 1,175 DETs consisting of 694 upregulated transcripts and 481 downregulated transcripts were verified at D1. There were 1,817 transcripts expressed differently at D2, including 864 upregulated transcripts and 1,235 downregulated transcripts. A total of 1,916 DETs were identified at D3, consisting of 681 upregulated transcripts and 1,235 downregulated transcripts (Figure 5B). The largest number of DETs was identified at D4. There were 3,712 DETs, including 1,120 upregulated transcripts and 2,592 downregulated transcripts.

### Functional Characterization of Cold-Responsive Differentially Expressed Transcripts in Rice Anthers

**FIGURE 6:**
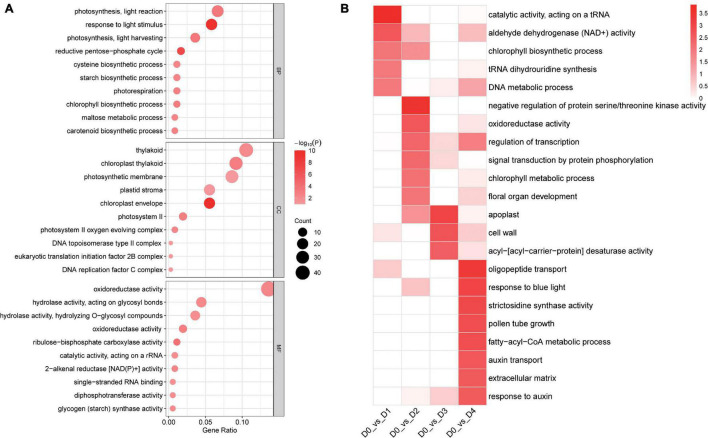
**(A)** Gene Ontology enrichment among the DETs between low-temperature groups from different timepoints and the control group **(B)** Enrichment for GO of 561 common DETs found between the four low-temperature groups and the control group.

A total of 561 common DETs were found between the four low-temperature groups and the control group, after enrichment with TopGO (Figure 5C). According to gene ontology (GO) enrichment analysis, the DETs were enriched mainly in groups corresponding to the chloroplast envelope (20 transcripts), response to light stimulus (21 transcripts), photosynthesis, light reaction (24 transcripts), and oxidoreductase activity (7 transcripts) (Figure 6A).

Comparing DETs enriched in D1 vs. D0, 185 specific DETs were enriched into five GO terms. The most enriched terms were: catalytic activity (acting on a tRNA), aldehyde dehydrogenase (NAD +) activity, chlorophyll biosynthetic process, tRNA dihydrouridine synthesis, and DNA metabolic process. 352 specific DETs enriched in D2 vs. D0 were enriched in 11 GO terms. The most enriched terms were: negative regulation of protein serine/threonine kinase activity, oxidoreductase activity (acting on the CH-CH group of donors), regulation of transcription (DNA-templated), signal transduction by protein phosphorylation, chlorophyll metabolic process, and floral organ development. 218 specific DETs enriched in D3 vs. D0 were enriched in three GO terms. The most enriched terms were: apoplast, cell wall, and acyl-[acyl-carrier-protein] desaturase activity. 1,973 specific DETs enriched in D4 vs. D0 were enriched into 32 GO terms. The most enriched terms were oligopeptide transport, response to blue light, strictosidine synthase activity, pollen tube growth, fatty-acyl-CoA metabolic process, auxin transport, extracellular matrix, and response to auxin (Figure 6B).

**FIGURE 7:**
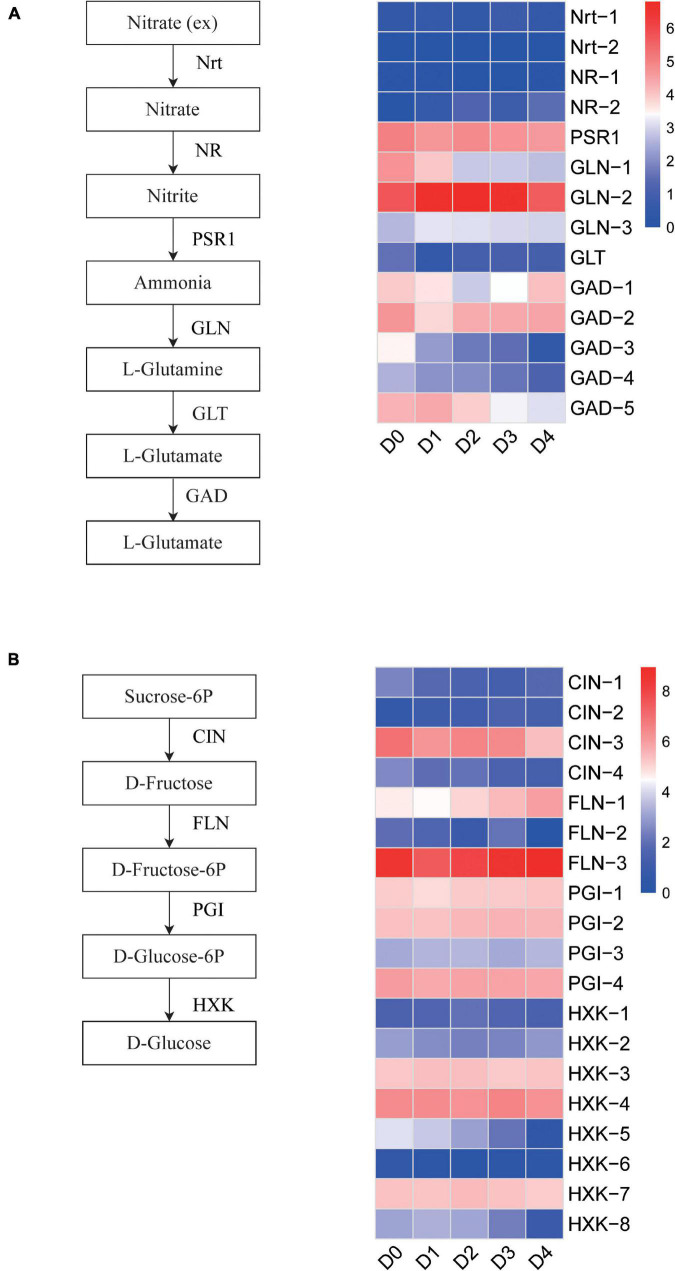
**(A)** Heatmap and metabolic pathway of glutathione metabolism between low-temperature groups and the control group. **(B)** Heatmap and metabolic pathways of sugar metabolism between low-temperature groups and the control group.

To understand the transcript expression profile of glutathione and sugar metabolism in response to cold stress, we used the Kyoto Encyclopedia of Genes and Genomes (KEGG) pathway database as a reference to show the DET expression patterns of glutathione and sugar metabolism in the rice anther. Glutamate decarboxylase (GAD) catalyzes the conversion of L-glutamate to γ-aminobutyric acid (GABA). Substantial conversion of glutamate to GABA was found to be proportional to the degree of cold stress. Glutamate decarboxylase transcripts Os04t0447400-01 and Os04t0447800-01 also showed a gradual downregulation in nitrogen metabolism. The TPMs of Os04t0447400-01 were 3.64, 2.23, 1.82, and 0.39 in the D1–D4 sample, respectively. The TPMs of Os04t0447800-01 were 3.23, 2.93, 2.06, and 1.21 in the D1–D4 sample, respectively (Figure 7A). The hexokinase transcripts Os05t0187100-01 and Os07t0446800-00 showed a gradual downregulation in sugar metabolism. The TPMs of Os05t0187100-01 were 12.32, 7.06, 3.28, and 0.49 in the D1–D4 samples, respectively. The TPMs of Os07t0446800-00 were 8.92, 7.64, 4.09, and 0.92 in the D1–D4 samples, respectively (Figure 7B).

### Identification of Co-expression Network Modules in Response to Cold Pressures

**FIGURE 8:**
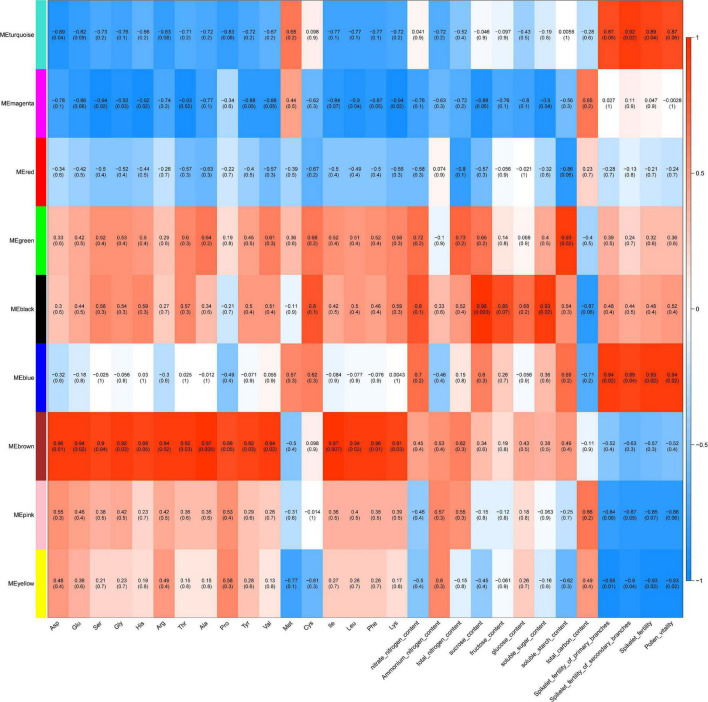
Module-trait relationship heatmap for different traits and gene modules. Gene modules are denoted by an arbitrary color name. Bins show the Pearson correlation coefficient value between gene expression levels of each module within traits and *P*-values. A value of 1 (red) and –1 (blue) both quantify the strongest correlation, and 0 (white) quantifies no correlation.

To identify the DEGs associated with the different phenotypes, all DETs were pooled together and used for WGCNA. A total of 5,129 DETs (D0_vs_D1, D0_vs_D2, D0 vs. D3, D0 vs. D4, D1 vs. D2, D2 vs. D3, D3 vs. D4) were used for the analysis (Figure 5A and Supplementary Figure 2). Soft threshold power and connectivity between the DETs were screened prior to the WGCNA module analysis. A soft threshold power of 20 was selected according to the preconditions of the approximate scale-free topology. Accordingly, a total of nine WGCNA modules were identified, including 31(magenta) to 1,876 (turquoise) transcripts in each module (Supplementary Figure 3). Amino acid, sugar, and spike phenotypes were associated with module results, and magenta, blue, brown, and yellow modules were significantly (cor > 0.5 or cor < −0.5, *P* < 0.05) correlated with different phenotypes (Figure 8).

### Modules Associated With Cold Stress-Related Phenotype

The brown module (719 transcripts) (Supplementary Figure 4) was significantly and positively correlated with the concentrations of 14 amino acids (cor > 0.5, *P* < 0.05), including Asp, Glu, Ser, Gly, His, Arg, Thr, Ala, Tyr, Val, Ile, Leu, Phe, and Lys (Figure 8). DETs in the brown module were mainly associated with photosynthesis (light harvesting in photosystem I), the chloroplast envelope, plastoglobuli, protein-chromophore linkage, the reductive pentose-phosphate cycle, the thylakoid, oxidoreductase activity (acting on the CH-CH group of donors, NAD or NADP as acceptor), and carotenoid biosynthetic processes. The magenta module (31 transcripts) (Supplementary Figure 5) was significantly and negatively correlated with the concentrations of 7 amino acids (Ser, Gly, His, Thr, Val, Leu, and Lys), sucrose content, and soluble sugar content (cor < −0.5, *P* < 0.05) (Figure 8). DETs in the magenta module were mainly associated with the thiamine metabolic process and beta-galactosidase activity. The blue module (1,040 transcripts) (Supplementary Figure 6) was significantly and positively correlated with the spikelet fertility of primary branches, spikelet fertility of secondary branches, overall spikelet fertility, and pollen vitality (Figure 8). DETs in the blue module were mainly associated with integral components of the plasma membrane, SCF ubiquitin ligase complex, protein ubiquitination, phenylpropanoid biosynthetic processes, sugar transmembrane transporter activity, ubiquitin protein ligase activity, polysaccharide catabolic processes, hydrolase activity (hydrolyzing O-glycosyl compounds), and ubiquitin-protein transferase activity. GO enrichment analysis suggested that ubiquitination and transmembrane transport play important roles in the regulatory network in response to cold stress. In eukaryotic cells, the ubiquitin proteasome system (UPS) determines protein turnover in response to various external stimuli. E3 ubiquitin-protein ligase (ARI5, ARI7, ARI9, RHA2B, XBOS36, and UPL4), which is a part of the UPS, is a regulator involved in stress-responsive pathways. RING-H2 finger protein (ATL45, ATL46, ATL56, and ATL49) acts as a protein–protein interaction domain and is necessary to catalyze the E3 ligase activity of RING-finger proteins. Potassium channel (KAT4 and KAT6), cyclic nucleotide-gated ion channel 4(MDK4.7), chloride channel protein CLC-c, and cyclic nucleotide-gated ion channel 17, are related to transmembrane transport in response to cold stress. To find hub transcripts under cold stress, we constructed a network of blue modules. The hub transcripts in the blue module network include Os02t0219000-01, which is an interferon-related developmental regulator protein homologous to the At1g27760 gene in *Arabidopsis*; Os01t0218350-00, a tetratricopeptide repeat-containing thioredoxin; Os08t0197700-00, similar to luminal-binding protein 5; and Os11t0200000-01, which is histone deacetylase 19 (Supplementary Figure 8).

The yellow module (706 transcripts) (Supplementary Figure 7) was significantly and positively correlated with the spikelet fertility of primary branches, the spikelet fertility of secondary branches, overall spikelet fertility, and pollen vitality (Figure 8). DETs in the yellow module were mainly associated with DNA replication, DNA binding, response to abscisic acid, response to osmotic stress, N-acylphosphatidylethanolamine-specific phospholipase D activity, and cold acclimation. ABA is an isoprenoid phytohormone that regulates various physiological processes ranging from stomatal opening to protein storage and provides adaptation to cold stress. Ten genes were found in the yellow module response to abscisic acid, including water stress-inducible protein Rab2, abscisic acid receptor PYL4, calcium-dependent protein kinase 11, two galactinol-sucrose galactosyl transferases, two dehydrins, EID1-like F-box protein 3, DELLA protein GAI, and embryonic abundant protein 1. The hub transcripts in the yellow module network include Os03t0758700-01, a WD40 repeat domain-containing protein, and Os06t0220500-01(7-deoxyloganetin glucosyltransferase). Pacbio.T01382 is a new transcript of sucrose synthase 1, and sucrose synthase (SUS) gene expression is induced in response to environmental stress. Os01t0172400-01, which is similar to phospholipase D alpha 1, is involved in the response of plants to salt stress. Os01t0261200-01 is NAC domain-containing protein 74, and NAC family genes play a key role in the abiotic stress response network (Supplementary Figure 9).

### Protein Interaction Analysis of Hub Transcripts in the Blue and Yellow Modules

In the PPI network constructed from comparing hub transcripts to the STRING database, no significant interactions were found in the blue modules. In the yellow modules, the DNA replication licensing factor MCM 4 interacted with four genes: minichromosome maintenance protein 10 (Os09t0539400-00), and three transducin family proteins: Os03t0758700-01, Os07t0486000-01, and Os08t0163100-01. These results indicate that mini-chromosome maintenance proteins may play an important role in DNA replication induced by cold stress.

### Validation of Differentially Expressed Genes in RNA-Seq by Quantitative Reverse Transcription Polymerase Chain Reaction

We compared the log_2_ fold change of 16 selected DEGs between RNA-Seq and qRT-PCR to validate the results of RNA-Seq, demonstrating a correlation coefficient (*R*^2^) of 0.685 (Supplementary Figure 10). The qRT-PCR data were consistent with the RNA sequencing data, indicating the reliability of the RNA-Seq results.

## Discussion

### Low Temperature Stress Decreased Pollen Viability and Spikelet Fertility by Affecting Carbon and Nitrogen Metabolism of the Anthers

Rice is a staple food for over half of the world’s population (Boliko, 2019), and LTs are an important factor restricting rice production, especially in northeast China, Korea, and Japan during the reproductive stage (Zhang et al., 2017). Our study addresses several gaps in the literature concerning the mechanisms of the response of rice plants to LT stress. Current studies on the effects of LT stress on rice during the reproductive stage have mainly focused on leaves and panicles at the booting (1–2 cm young panicles) and flowering stages, although there are a few studies which have previously examined the effects of LT stress on anthers at the meiosis stage, or performed research on the morphology and microstructure of rice anthers (Jia et al., 2015; Tian et al., 2015; Zeng et al., 2016, 2017; Siddik et al., 2019; Susanti et al., 2019).

In addition, most studies concerning the metabolism of carbon and nitrogen in anthers have, at present, focused on the effects of high-temperature stress (Rezaul et al., 2019; Santiago and Sharkey, 2019; Baslam et al., 2021). It has been found that under heat stress, the sugar transport process of the anther is impeded while the content of sucrose and starch decreases, whereas fructose and glucose content increases (Lee et al., 2020; Kumar et al., 2021). Comparing this observation to the results of the few studies investigating LT stress have shown that LT stress results in the accumulation of sucrose in rice anthers, accompanied by a reduction of starch in mature pollen (Oliver et al., 2005, 2007). Changes in amino acid synthesis and accumulation throughout anther development have been shown to be necessary for maintaining male reproductive ability. Contributing to this knowledge, our study showed that there were significant changes in nitrogen and sugar contents over time in rice anthers at the meiosis stage experiencing LT stress. At D1, the sucrose, fructose, glucose, soluble sugar, soluble starch, nitrate nitrogen, ammonium nitrogen, and total nitrogen content had significantly increased. However, at D4, the soluble starch content decreased significantly, while the glucose, ammonium nitrogen, and total nitrogen level increased significantly, and other differences were not significant. Previous studies have found that the activities of wheat acid invertase and SUS decreased under drought stress (Nemati et al., 2018). High-temperature stress contributes to the decreasing activity of cell wall invertase in pollen (Jain et al., 2010). LT inhibits the expression of OsINV4 and OsMST8 in the tapetum, leading to disruption of starch formation during anther development (Oliver et al., 2005; Mamun et al., 2006). Therefore, we speculate that the decrease in rice anther enzyme activity under long-term LT stress leads to the interruption of starch synthesis and at the same time, causes more carbon skeletons to participate in nitrogen metabolism, which also explains the reason for the increase in nitrogen content (Asthir et al., 2013). In addition, the anther protein is degraded under cold stress, and the amino acid composition changes significantly (Ma et al., 2020). We found that concentrations of 15 of the 17 essential amino acids increased, especially Asp, Glu, Arg and Tyr, and the general trend increased at first, before then decreasing, which is almost consistent with the trend of carbohydrate content. Met and Cys levels decreased and were the lowest at D4.

With an increase in the number of days of LT stress, spikelet fertility and pollen vitality gradually decreased. Through multiple linear stepwise regression and path analysis we found that Pro, Ala, Asp, soluble starch content, and total nitrogen content of the anther are the key factors to determine spikelet fertility and pollen vitality during LT stress.

### Differentially Expressed Transcripts Expression Patterns of Glutathione and Sugar Metabolism in Rice Anther During Low Temperature Stress

RNA-Seq and Iso-Seq are powerful tools for identifying transcriptomic changes in the response mechanisms of rice to cold stress. Our study provides a comprehensive transcriptome survey to understand the transcripts responsive to LT stress at the anther stage in rice. This contributes to knowledge provided by previous studies which identified several key genes that control starch and amino acid synthesis in rice (Oliver et al., 2005; Kong et al., 2007; Mu et al., 2009; Li T. et al., 2010; Lee et al., 2020), and extensive knowledge regrading the leaves at the seedling and reproductive stages to create a more complete picture of the mechanisms controlling rice growth.

There are few studies on the molecular mechanisms of sugar metabolism and amino acid synthesis in rice anthers in response to LT stress during meiosis stage. In our study, we provide a comprehensive analysis of changes in important metabolic pathways in rice.

Glutathione and sugar metabolism are important for plants to resist abiotic stress, and a large amount of energy is required in this process (Zechmann, 2014; Khan et al., 2020). Hexokinase, the key rate-limiting enzyme of plant respiration and glycolysis metabolism, plays a vital role in cold response through plant sugar sensing and sugar signal transduction (Wang et al., 2019). The hexokinase transcripts Os05t0187100-01 (OsHXK7) and Os07t0446800-00 (OsHXK1) showed a gradual decrease from D0 to D4 in sugar metabolism. Under cytoplasmic hexokinase hypoxia, OsHXK7 may play a unique role in rice sugar metabolism and seed germination by enhancing glycolysis-mediated fermentation (Kim H. B. et al., 2016). OsHXK7 is downregulated by exogenous application of glucose and fructose (Wang et al., 2017). Therefore, the downregulation observed in our study may be related to the increase in glucose content during LT stress. OsHXK1 is the target of methylation modification by AGO protein OsAGO2 and plays an important role in the development of the anther tapetum. Overexpression of OsHXK1 leads to excessive accumulation of reactive oxygen species in the tapetum, and premature initiation of PCD and pollen abortion (Zheng et al., 2019). PCD of the tapetal layer is delayed during LT (Oliver et al., 2005), so we can speculate that this may be related to the downregulation of OsHXK1.

Glutamate decarboxylase catalyzes the conversion of L-glutamate to GABA. GABA appears to impart partial protection to various abiotic stresses in most plants by increasing leaf turgor, increasing osmolytes and reducing oxidative damage by stimulation of antioxidants (Ramos-Ruiz et al., 2019). In our study, Os04t0447400-01 (similar to OsGAD2) and Os04t0447800-01 (OsGAD2) showed a gradual down regulation during LT. OsGAD2 is actually Ca^2+^ /CaM-independent with respect to enzyme activation, and the C-terminal extension of OsGAD2 acts as an autoinhibitory domain (Akama and Takaiwa, 2007). The effect of the C-terminal extension autoinhibitory domain of OsGAD2 during LT stress requires further study.

### Prediction of Key Genes and Proteins in Response to Low Temperature Stress

The key genes and proteins in response to stress include the ABA pathway and the ABA-independent pathway. The hub transcripts in the blue module network included two ABA pathway genes, including an AtSAT32 analog (which in *Arabidopsis* is more sensitive to salt stress and reduces fertility) (Park et al., 2009), and a tetratricopeptide repeat-containing thioredoxin (TTL; required for osmotic stress tolerance and male sporogenesis in *Arabidopsis*). TTL1 is a positive regulator of ABA signaling under stress (Rosado et al., 2006). TTL2 performs a distinct function among the TTLs in male gametophyte development, and is involved in male gametophytic transmission (Lakhssassi et al., 2012). The hub transcripts in the blue module network also included two ABA-independent pathway genes, an OsBiP5 analog (an ER stress-related BiP protein that is regulated by the OsIRE1/OsbZIP50 pathway) (Wakasa et al., 2012), and histone deacetylase 19. HDAC genes are both tissue/organ-specific, and most of them are responsive to drought or salt stress (Hu et al., 2019). The hub transcripts in the yellow module network include an *Arabidopsis* At1g69400 gene homolog, which is a pivotal regulatory factor in the process of plant development and stress signaling transmission (Mishra et al., 2012; Kim et al., 2021); 7-deoxyloganetin glucosyltransferase, which is most expressed in anthers, plays a major role in cell homeostasis and may contribute to cell chemical stability and reduce chemical activity under stress conditions (Vogt and Jones, 2000); Pacbio.T01382, a new transcript of SUS1, which is induced in response to environmental stress (Stein and Granot, 2019); and phospholipase D alpha 1 analog, which is involved in salt tolerance through the mediation of H + -ATPase activity and transcription (Shen et al., 2011). These hub transcripts in LT stress need to be continued to be studied in depth.

The response to LT stress is a multi-gene regulatory network, in which many transcription factors play an important role in regulating the expression of stress-induced genes (Takasaki et al., 2010; Lan Thi Hoang et al., 2017).

The hub transcripts in the yellow module network also included transcription factors. Os01t0261200-01 is NAC domain-containing protein 74 (OsNTL3; OsNAC8). OsNTL3 directly binds to the OsbZIP74 promoter and regulates its expression in response to heat stress. In turn, up-regulation of OsNTL3 by heat stress is dependent on OsbZIP74 (Liu et al., 2020).

In our further investigation, we found that the DNA replication licensing factor MCM 4 interacted with transducin family proteins and minichromosome maintenance proteins (MCM). MCM proteins ensure that DNA is replicated only once per cell division cycle (Gill et al., 2021). Overexpression of pea MCM6 was found to confer high salt tolerance to tobacco, leading to better growth, photosynthetic activity, and minimal reduction in yield, most likely by activating the expression of stress-related genes (Dang et al., 2011; Brasil et al., 2017). Therefore, MCM 4 protein may play an important role in DNA replication induced by cold stress.

## Conclusion

In our study, LT stress decreased pollen viability and spikelet fertility by affecting the sugar, nitrogen and amino acid contents of the anthers. We performed RNA-seq and ISO-seq experiments to study the genome-wide expression profiles in these LT stress anthers. Hexokinase and glutamate decarboxylase participating in starch and sucrose metabolism may play important roles in the response to cold stress. Using WGCNA, nine co-expression gene modules were calculated and we identified transcripts involved in ABA and ubiquitination pathways in the blue and yellow modules, which indicates that these transcripts can improve cold tolerance throughout the meiosis period of rice. In the PPI network, MCM4 protein may play an important role in DNA replication induced by cold stress. Finally, our findings provide valuable insights into the molecular and genetic mechanisms responsible for cold tolerance in rice.

## Data Availability Statement

The datasets presented in this study can be found in online repositories. The names of the repository/repositories and accession number(s) can be found below: https://www.ncbi.nlm.nih.gov/, PRJNA772921, https://www.ncbi.nlm.nih.gov/, PRJNA773058.

## Author Contributions

ZQ: writing original draft, investigation, and methodology. YJ: funding acquisition, investigation, and methodology. YD and HC: investigation and methodology. XW, HlZ, HL, and JW: project administration. DZ and HwZ: writing, review and editing, supervision, and funding acquisition. All authors contributed to the article and approved the submitted version.

## Conflict of Interest

The authors declare that the research was conducted in the absence of any commercial or financial relationships that could be construed as a potential conflict of interest.

## Publisher’s Note

All claims expressed in this article are solely those of the authors and do not necessarily represent those of their affiliated organizations, or those of the publisher, the editors and the reviewers. Any product that may be evaluated in this article, or claim that may be made by its manufacturer, is not guaranteed or endorsed by the publisher.

## Supplementary Material

Supplementary Figure 1 Transcriptome correlations (Pearson’s correlation coefficients) between samples.

Supplementary Figure 2 Histogram of the DETs between Adjacent time point.

Supplementary Figure 3 Weighted Gene Co-expression Network Analysis (WGCNA) of the DETs.

Supplementary Figure 4 The expression patterns of the brown module are shown by the heatmap.

Supplementary Figure 5 The expression patterns of the magenta module are shown by the heatmap.

Supplementary Figure 6 The expression patterns of the blue module are shown by the heatmap.

Supplementary Figure 7 Gene Co-expression of the blue module are shown by the Cytoscape.

Supplementary Figure 8 The expression patterns of the yellow module are shown by the heatmap.

Supplementary Figure 9 Gene Co-expression of the yellow module are shown by the Cytoscape.

Supplementary Figure 10 16 DEGs between RNA-Seq and qRT-PCR.
