# Supplementary material for: Integrated Isoform Sequencing and Dynamic Transcriptome Analysis Reveals Diverse Transcripts Responsible for Low Temperature Stress at Anther Meiosis Stage in Rice
Source: Front Plant Sci. 2021 Dec 17;12:795834. doi: 10.3389/fpls.2021.795834 (PMC8718874; doi:10.3389/fpls.2021.795834)
Supplement: Supplementary file 2 [file Table_2.docx]

Table S2 Multiple linear stepwise regression analysis of spikelet fertility and pollen vitality under low temperature

| Dependent variables (Y) | Stepwise regression | Correlation coefficients (r) | R^2^ |
| --- | --- | --- | --- |
| Spikelet fertility(Y_SF_) | Y_SF_=138.224-0.009X_1_+1.562X_2_-7.891X_3_ | 0.959 | 0.920 |
| Pollen vitality(Y_PV_) | Y_PV_=114.703-0.016X_1_-0.457X_2_-13.068X_3_-0.022X_4_-0.005X_5_ | 0.996 | 0.992 |

X_1_: Pro, X_2_: soluble starch content, X_3_: total nitrogen content, X_4_: Ala, X_5_: Asp

Table S3 Path analysis of spikelet fertility under low temperature

| Index | Direct path coefficients | Indirect path coefficients | | | Decision coefficient |
| --- | --- | --- | --- | --- | --- |
|  |  | Pro | SS | TN | R^2^ |
| Pro | -0.840 | —— | 0.193 | -0.064 | 0.488 |
| SS | 0.730 | -0.222 | —— | -0.127 | 0.024 |
| TN | -0.290 | -0.184 | 0.321 | —— | 0.005 |

SS: soluble starch content, TN：total nitrogen content

Table S4 Path analysis of pollen vitality under low temperature

| Index | Direct path coefficients | Indirect path coefficients | | | | | Decision coefficient |
| --- | --- | --- | --- | --- | --- | --- | --- |
|  |  | Pro | SS | TN | Ala | Asp | R^2^ |
| Pro | -1.749 | —— | -0.068 | -0.128 | 2.212 | -0.953 | -0.66 |
| SS | -0.259 | -0.462 | —— | -0.256 | 1.717 | -0.338 | -0.277 |
| TN | -0.582 | -0.383 | -0.114 | —— | 1.602 | -0.667 | -0.169 |
| Ala | 2.688 | -1.439 | -0.165 | -0.347 | —— | -1.059 | -8.96 |
| Asp | -1.177 | -1.416 | -0.074 | -0.330 | 2.419 | —— | -0.024 |

SS: soluble starch content, TN：total nitrogen content
