# Supplementary figures and images for: Integrated Isoform Sequencing and Dynamic Transcriptome Analysis Reveals Diverse Transcripts Responsible for Low Temperature Stress at Anther Meiosis Stage in Rice
Source: Front Plant Sci. 2021 Dec 17;12:795834. doi: 10.3389/fpls.2021.795834 (PMC8718874; doi:10.3389/fpls.2021.795834)

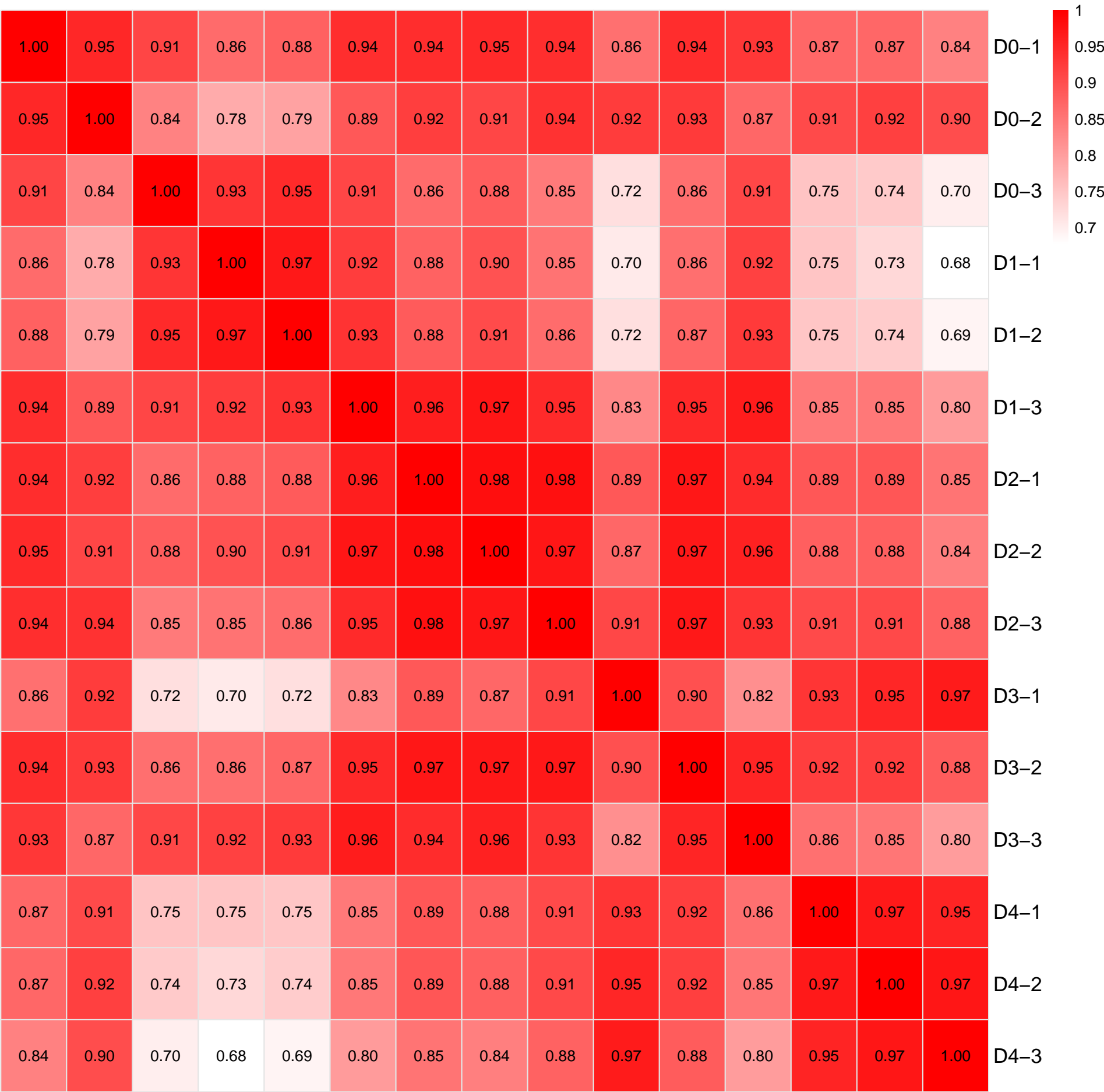

Supplement: Supplementary Figure 1 — Transcriptome correlations (Pearson’s correlation coefficients) between samples. [file Image_1.PDF]

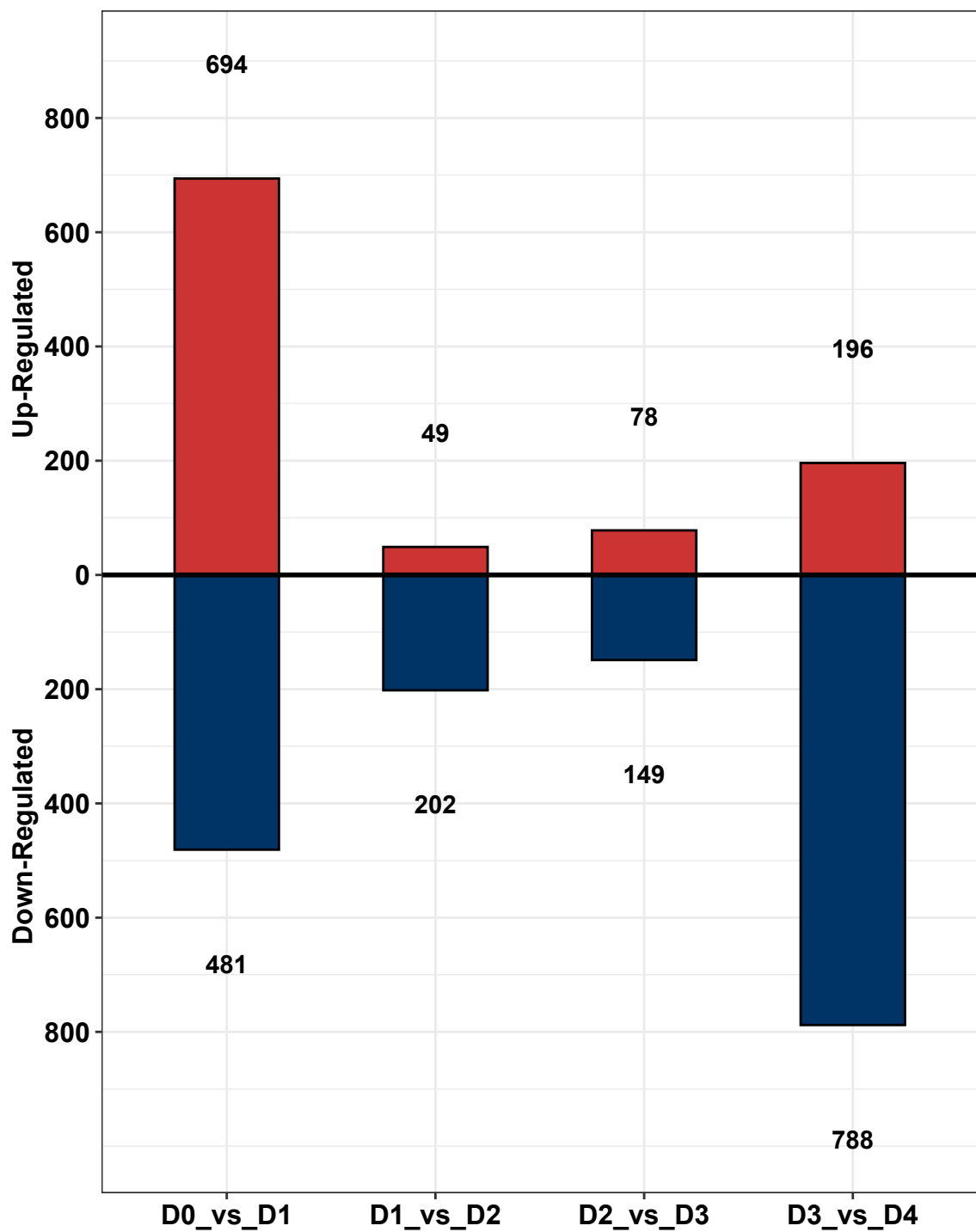

Supplement: Supplementary Figure 2 — Histogram of the DETs between Adjacent time point. [file Image_2.PDF]

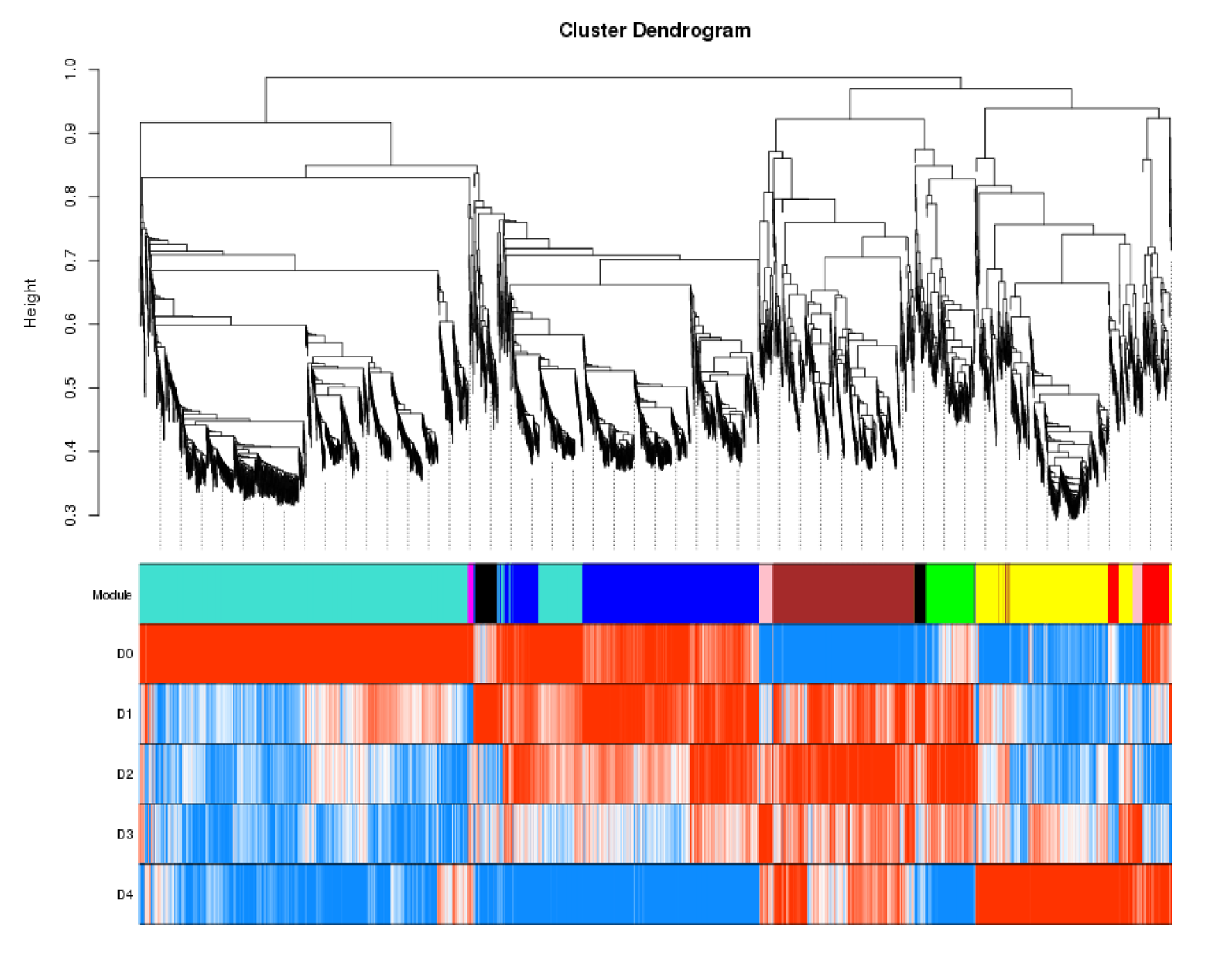

Supplement: Supplementary Figure 3 — Weighted Gene Co-expression Network Analysis (WGCNA) of the DETs. [file Image_3.TIF]

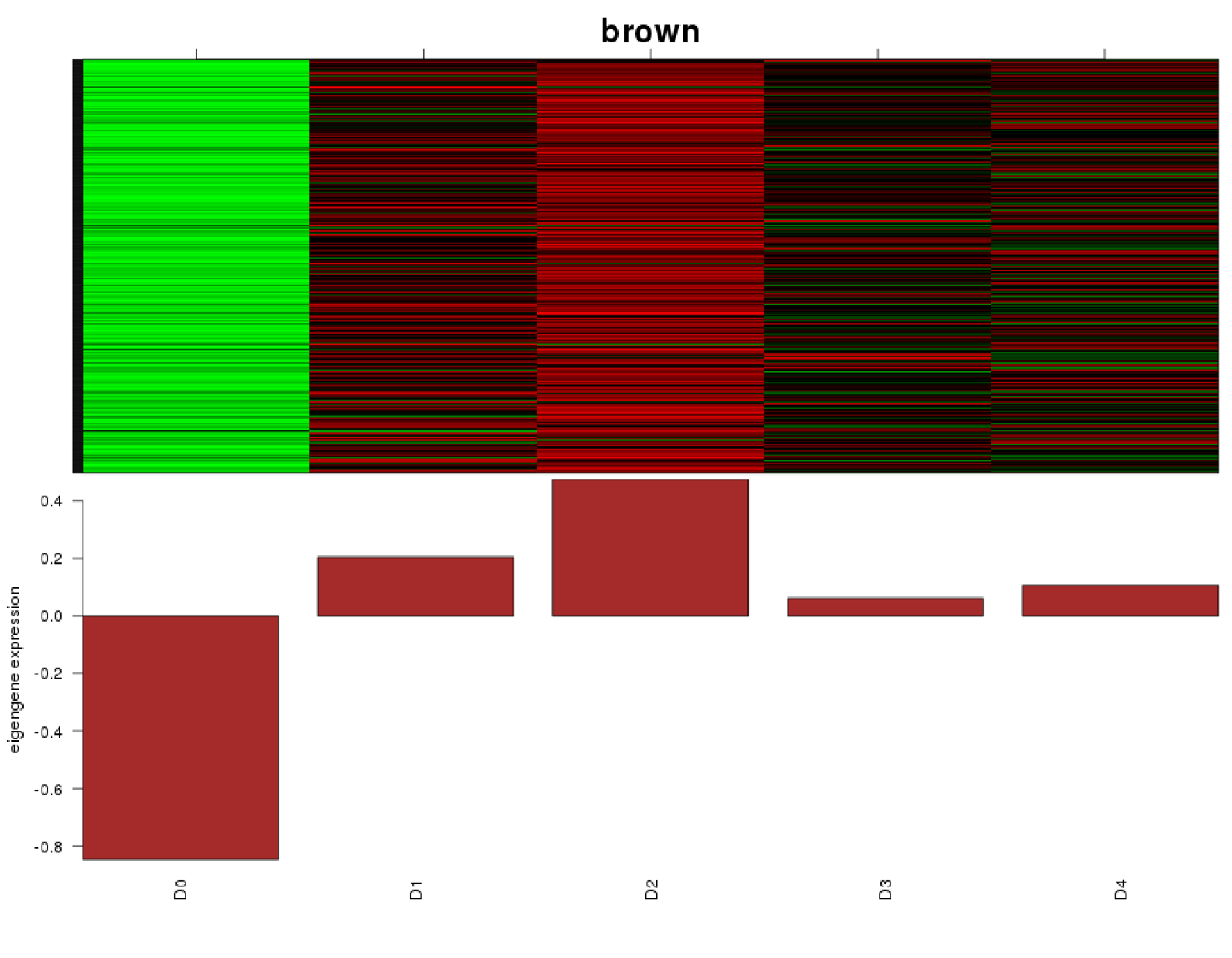

Supplement: Supplementary Figure 4 — The expression patterns of the brown module are shown by the heatmap. [file Image_4.TIF]

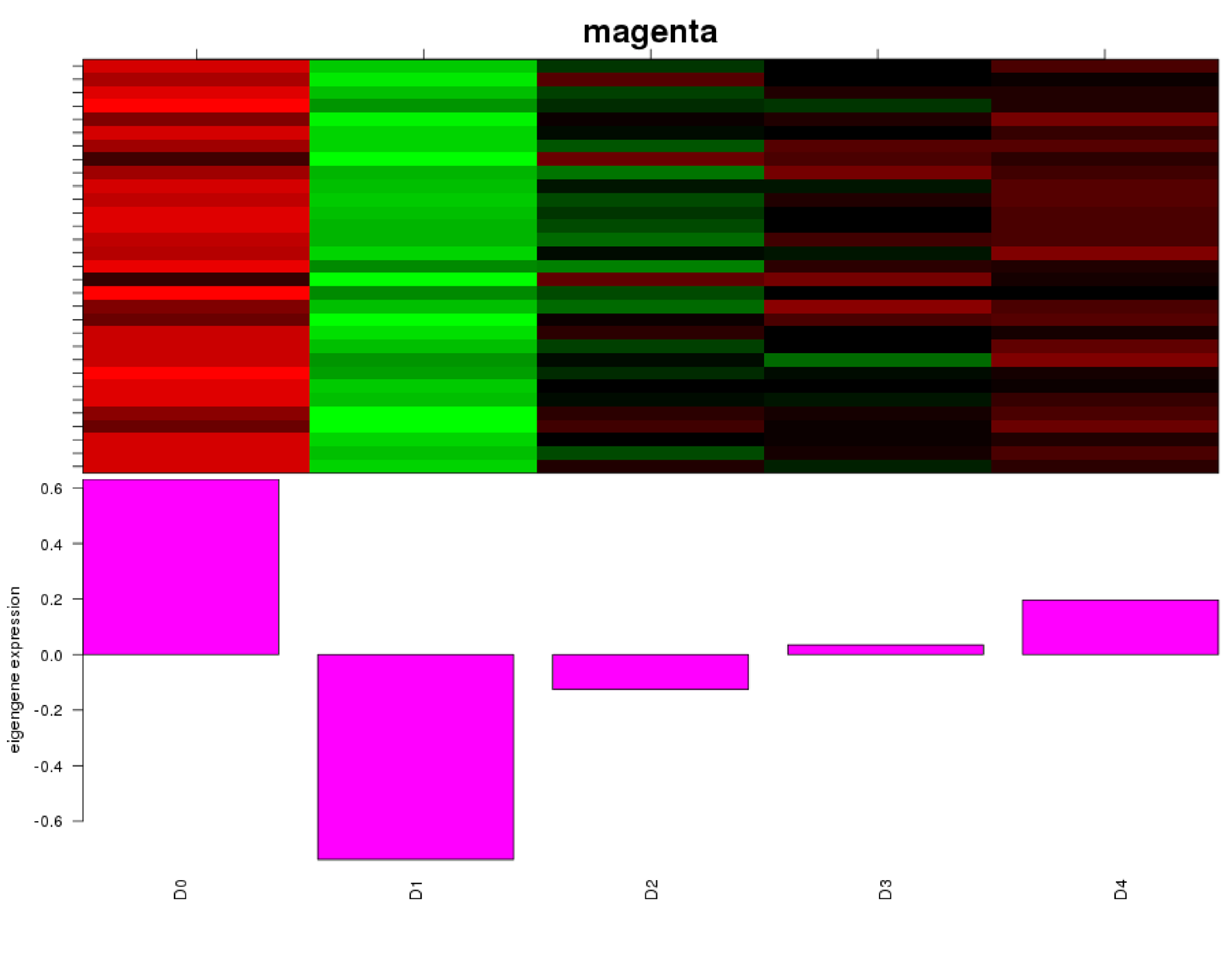

Supplement: Supplementary Figure 5 — The expression patterns of the magenta module are shown by the heatmap. [file Image_5.TIF]

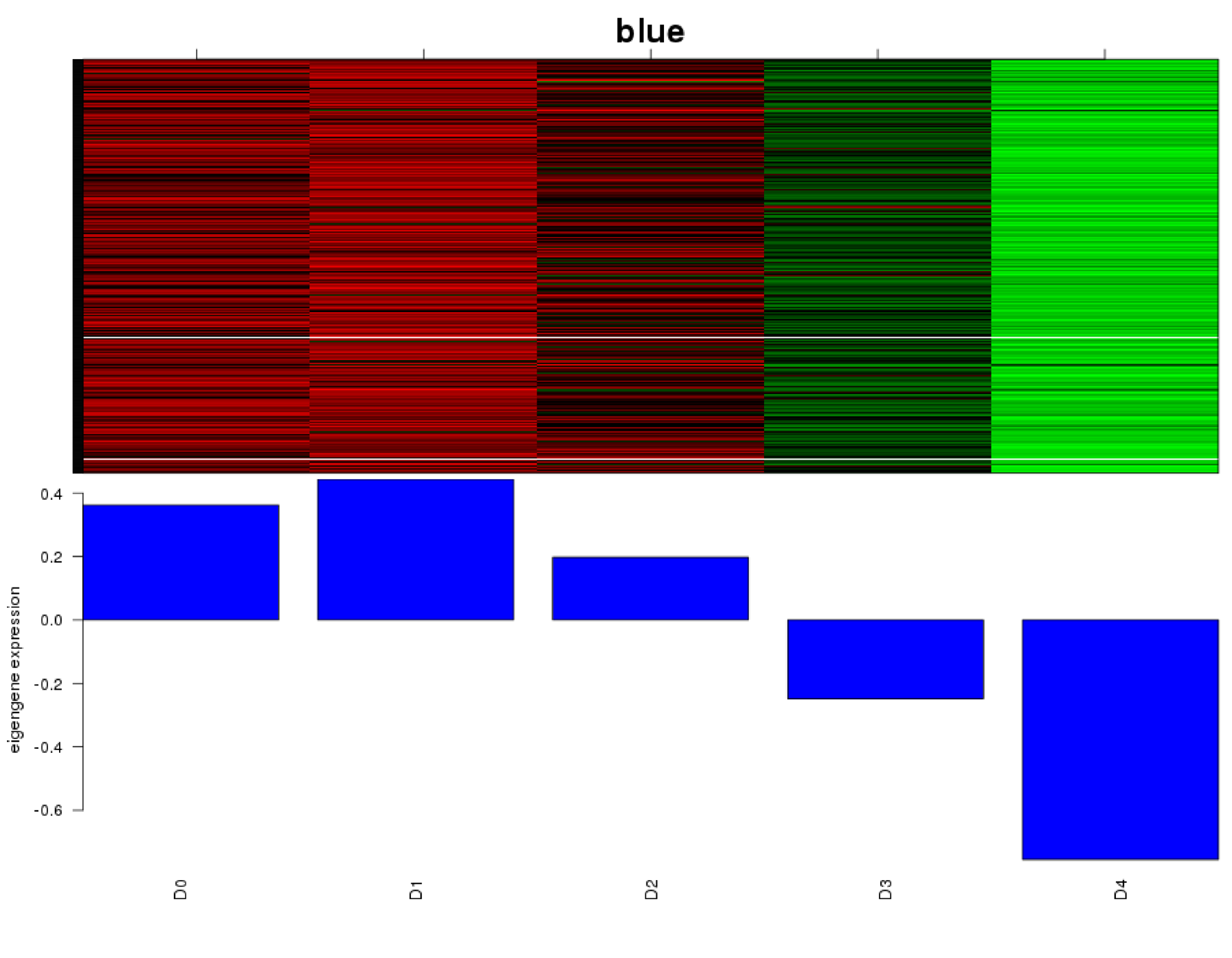

Supplement: Supplementary Figure 6 — The expression patterns of the blue module are shown by the heatmap. [file Image_6.TIF]

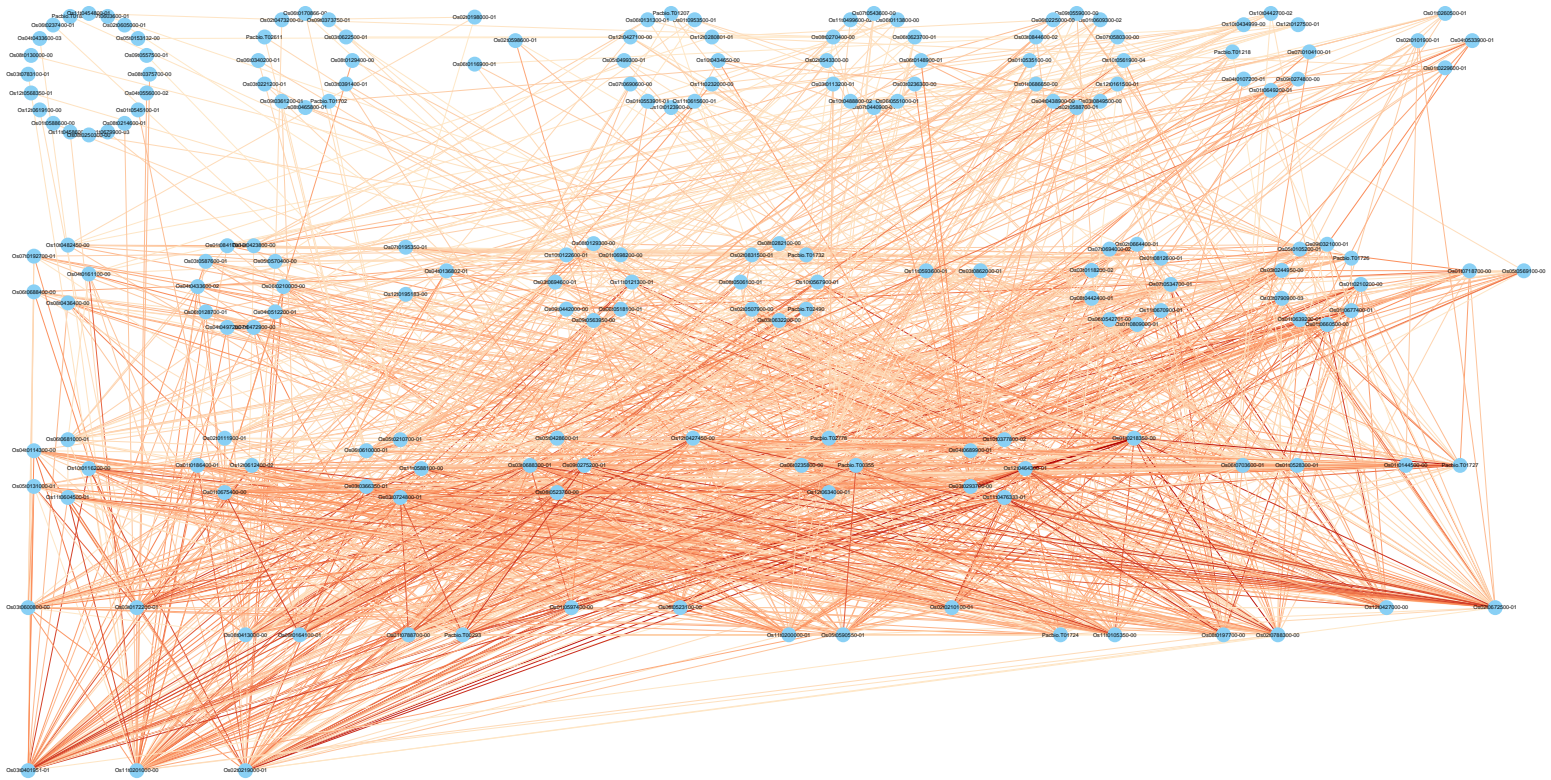

Supplement: Supplementary Figure 7 — Gene Co-expression of the blue module are shown by the Cytoscape. [file Image_7.pdf]

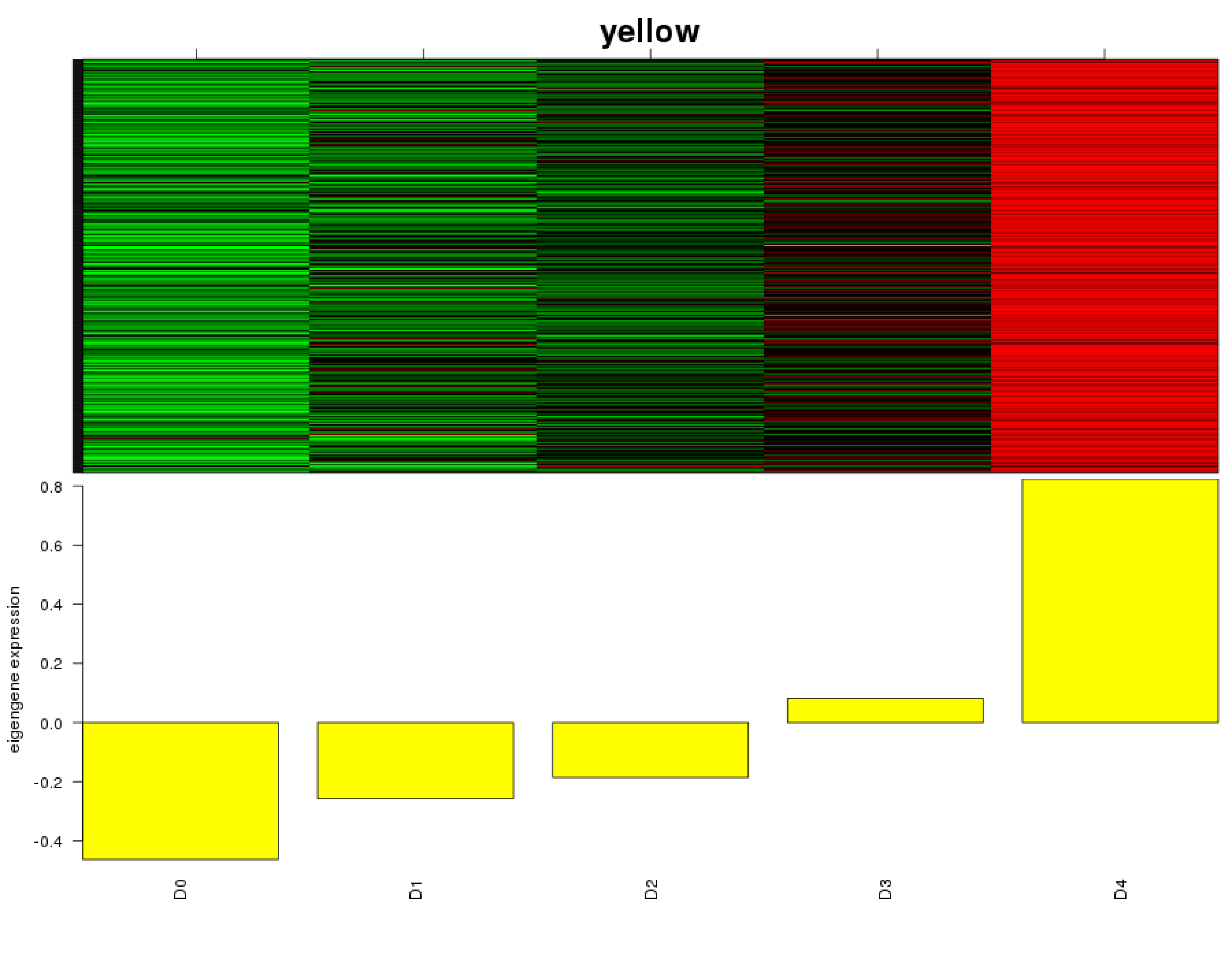

Supplement: Supplementary Figure 8 — The expression patterns of the yellow module are shown by the heatmap. [file Image_8.tif]

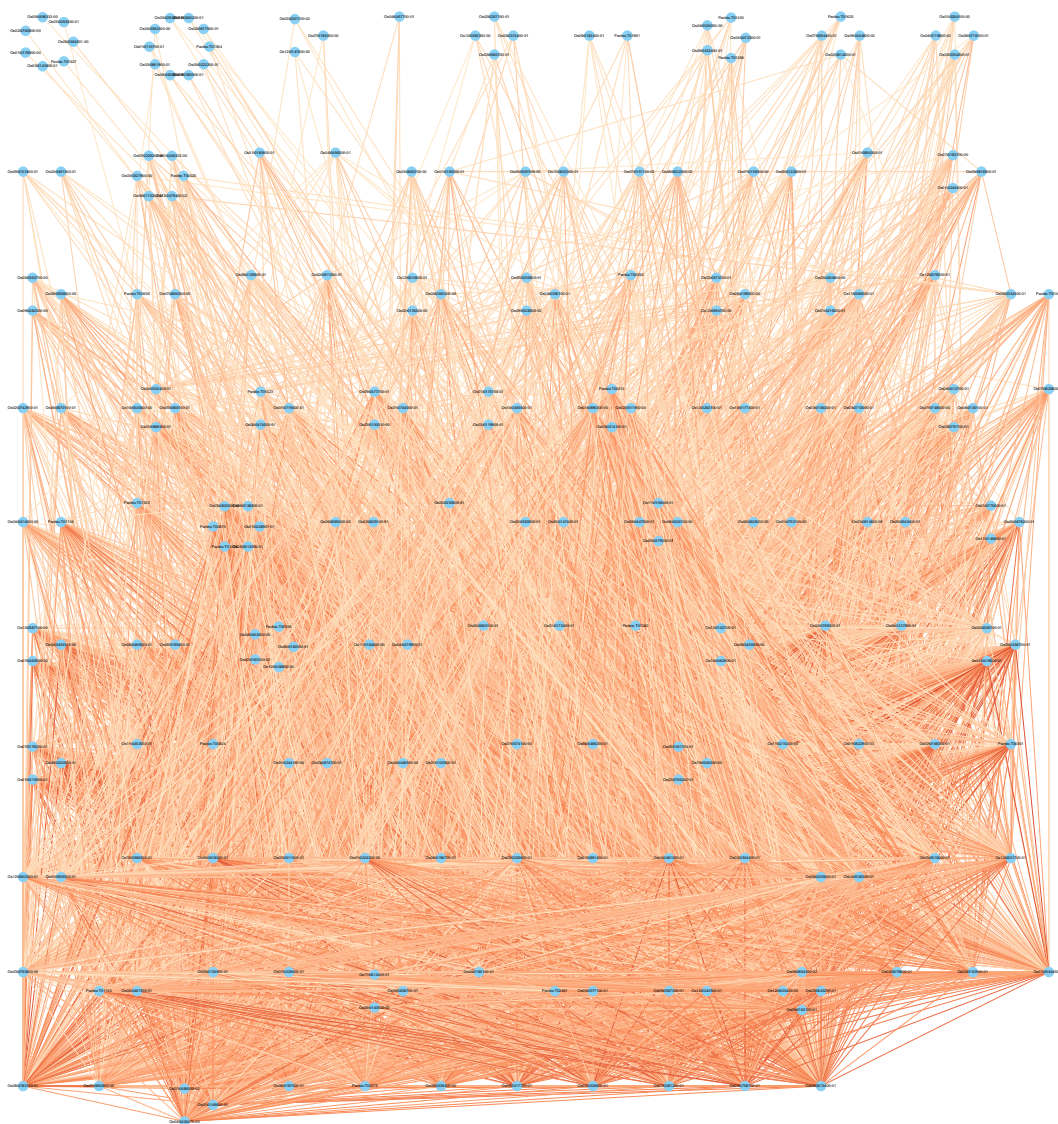

Supplement: Supplementary Figure 9 — Gene Co-expression of the yellow module are shown by the Cytoscape. [file Image_9.PDF]

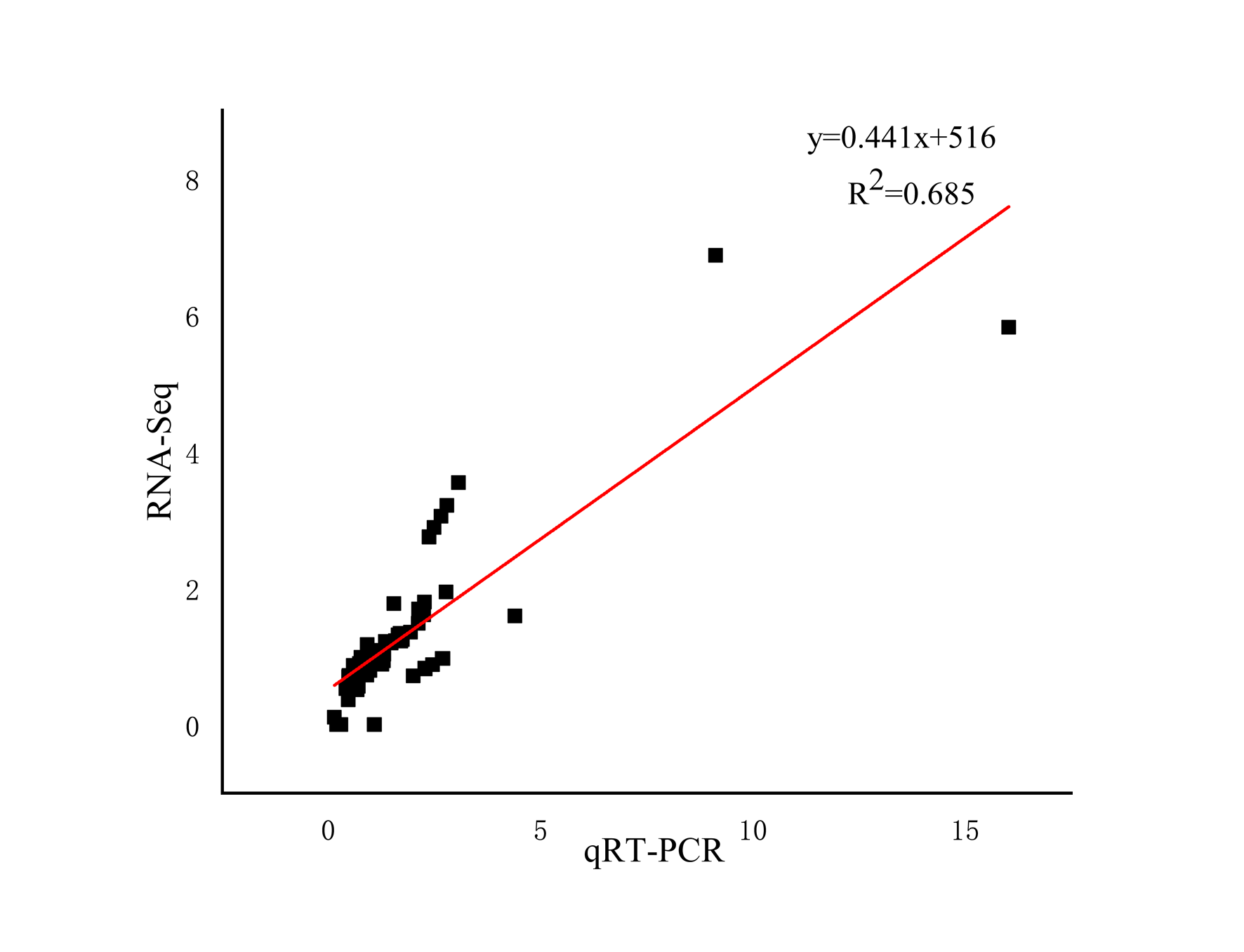

Supplement: Supplementary Figure 10 — 16 DEGs between RNA-Seq and qRT-PCR. [file Image_10.TIF]
